# Design and Evaluation of Wearable Solar Radiation Shields for Enhanced Personal Heat Exposure Monitoring

**DOI:** 10.3390/s25030945

**Published:** 2025-02-05

**Authors:** Dana Habeeb, Rahul Devajji, Nick Polak, Greatzel Unabia, Oscar Urbina

**Affiliations:** 1 Luddy School of Informatics, Computing, and Engineering, Indiana University, Bloomington, IN 47408, USA; rdevajji@iu.edu; 2 O’Neill School of Public and Environmental Affairs, Indiana University, Bloomington, IN 47405, USA; napolak@iu.edu; 3 School of Science and Technology, Georgia Gwinnett College, Lawrenceville, GA 30043, USA; infotech@greatzel.com; 4 College of Engineering, The University of Texas at El Paso, El Paso, TX 79902, USA; orurbina@miners.utep.edu

**Keywords:** wearable sensors, personal heat exposure, solar radiation shields, extreme heat

## Abstract

Extreme heat is one of the main climate-induced public health risks to communities around the world. Understanding an individual’s vulnerability to heat is challenging, as heat exposures vary significantly depending on occupation, travel behaviors, personal activities, and the surrounding urban environment. Previous validation studies have found that commonly used wearable temperature sensors are less reliable in highly urbanized areas and when worn in direct sunlight. The aim of our study is to investigate the potential to improve the reliability of wearable temperature sensors commonly used in personal heat exposure studies. To accomplish this aim, we designed and rapidly prototyped a set of solar radiations shields to decrease temperature bias when worn in direct sunlight and in areas of high impervious surfaces. In a field deployment, we tested four different form factors for solar radiation shields, which were specifically designed to house the iButton sensor and to be worn on-body. Initial results have shown that these wearable solar radiation shields can improve sensor reliability by decreasing temperature bias by 3 °F on average. These findings highlight the potential for wearable radiation shields to enhance personal heat exposure measurements in urban environments.

## 1. Introduction

Extreme heat in urban areas poses a serious public health risk, as it can lead to heat-related illnesses, exacerbate existing medical conditions, and disproportionately affect vulnerable populations such as the elderly, children and those with limited access to cooling resources. Determining heat vulnerability for urban residents is challenging. Heat exposures vary dramatically within the the built environment as does an individual’s susceptibility and adaptive capacity to heat. It is difficult to measure real-time and hyperlocal heat exposure especially at the personal level. When an individual checks the weather on their mobile devices, the data presented are typically captured by a single meteorological station located at the nearest airport [1,2], which can be tens of miles away from the individual. Unsurprisingly, temperature data from a single weather station in an urban area cannot account for variations in urban temperatures which can vary from 4 °F to as large as 22 °F [3,4,5].

Local environmental monitoring is a growing area of research that allows for more precise measurement of individuals’ exposure to extreme heat in urban environments. The deployment of urban environmental sensor networks, which use low-cost IoT technology, has increased, improving the ability to track temperature variations and heat indices in cities [6,7,8]. However, while these networks are becoming more common, they are not yet being used to provide real-time, hyperlocal temperature data to urban residents. Additionally, their scale is often insufficient to accurately capture an individual’s personal heat exposure [9]. In addition to urban sensor networks, mobile air temperature monitoring campaigns (motorized and non-motorized) with vehicles, bikes, backpack, and helmet systems have been deployed to capture hyperlocal temperature variations. This research trend illustrates the robust application and deployments of sensing technology to better capture hyperlocal urban environmental parameters [10,11,12,13,14,15].

Measuring heat exposure at the personal level is crucial for helping people understand and respond to health risks in their daily lives [16]. Personal exposure varies not only due to the diverse urban landscape but also based on socioeconomic factors, occupation, and lifestyle. Recent advancements in wearable sensing technology have expanded research on individual environmental monitoring in cities [17]. Wearable sensors have been used to measure environmental factors like air pollution, noise, and heat stress, though heat stress is the least explored [10,17]. This technology can also help address environmental justice issues by highlighting the disparities faced by overburdened and overexposed communities.

The past decade has seen an increase in personal heat exposure studies [18] where wearable temperature sensors have been used to measure personal heat exposures for vulnerable population groups such as outdoor workers, elders, and athletes [19,20,21,22,23,24,25,26,27,28]. Unlike backpack and helmet systems, these sensors are selected for long-term deployments and for everyday wearability with different stakeholder groups. The iButton, Kestrel Drop, and Hobo Pendant are the most commonly used wearable temperature sensors for personal heat exposure studies. Notably, the iButton sensor is the most popular of the wearable sensors, as it is used three times more than the other sensing technologies [18,29]. Although personal heat exposure is an important area of research, sensors used in these types of wearable heat studies were not originally designed to be worn either on-body or in outdoor environments. As such, researchers have begun investigating the performance and reliability of wearable temperature sensors by comparing their performance to co-located meteorological stations and investigating optimal on-body sensor placement [30,31,32,33]. These studies have shown that sensors overheat in direct sunlight and when deployed in areas with a high percentage of impervious surfaces. Overheating is due to a lack of shielding of direct sunlight, which is typically provided by solar radiation shields. Terrando et al. [34] and Bailey et al. [32] evaluated wearable temperature sensors and demonstrated that when housing wearable sensors inside a standard size solar radiation shield, shielding helps prevent overheating and improve sensor performance.

It is standard practice to house near-surface air temperature sensors inside solar radiation shields. However, these shields are not worn on the body due to their large size. Solar radiation shields vary in size with a range in diameter from 10 cm to 20 cm (4 to 8 inches), a range in height from 15 cm to 30 cm (6 to 12 inches), and can weigh as much as a kilogram. Instead, researchers will often use plastic cups as a substitute for solar radiation shielding [34] for personal exposure studies.

Solar radiation shields protect temperature sensors from overheating due to direct sunlight. These shields allow air to flow freely around the sensor while blocking sunlight, ensuring the measurements reflect the true ambient conditions. Most solar radiation shields have a layered or slatted structure, often called gills, resembling a stack of plates or rings. This design promotes air circulation while keeping the sensor shielded from solar radiation. Typically made from reflective, heat-resistant materials, such as white plastic, these shields are usually cylindrical or dome-shaped to enhance air flow and minimize heat absorption while also protecting the sensor from environmental elements.

Even though researchers have discussed the limitation of wearable sensors overheating in direct sunlight and have illustrated the importance of these wearable sensors being housed in solar radiation shields [29,32,34,35], they have not yet identified the need for designing and developing *wearable* solar radiation shields. To address this gap, we prototyped wearable 3D-printed solar radiation shields to improve the accuracy of wearable temperature sensors in personal heat exposure studies. We designed our shields specifically for iButton sensors due to their small size and frequent use in personal heat exposure studies. These shields can be attached to a belt or worn as earrings. We evaluated these wearable shields in a field study to determine whether they reduce temperature bias compared to unshielded iButtons. Finally, we conclude with a discussion regarding challenges in this field and opportunities moving forward to capture the potential of wearable sensing technology for both future personal heat exposure studies and for everyday wearability.

## 2. Materials and Methods

We designed and rapidly prototyped a series of 3D-printed wearable solar radiation shields to be used with on-body sensors. These shields can be attached to a belt or worn as earrings. The belt was chosen as a design location, as it is one of the most optimal locations for on-body sensors [18]. There are typically seven different on-body locations used for attaching wearable temperature sensors [18]. Sensors located on the belt have been shown to be one of the best locations as it reduces bias effects from dark clothing or perspiration. In contrast, earrings represent a new and unexplored location for capturing ambient temperatures in urban environments. To date, no studies have investigated this on-body location for ambient air temperature.

**Figure 1:**
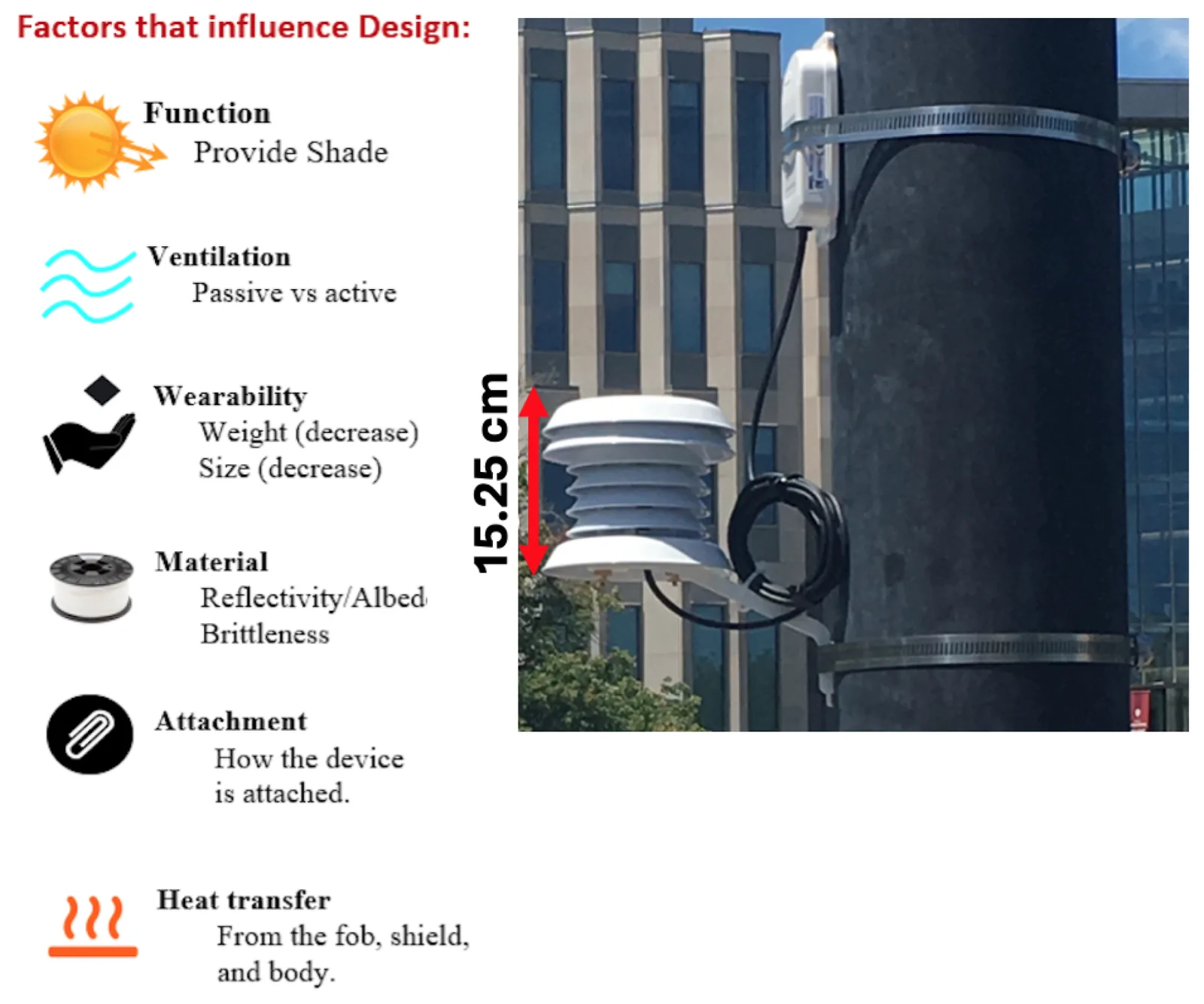
**Left**: A list of factors considered when prototyping *wearable* solar radiation shields. **Right**: A MX2302A Temperature/Relative Humidity Sensor (research grade) with standard size solar radiation shield.

When designing our wearable shields, we considered several key factors, including the shading and ventilation of the sensor, wearability, material type, attachment, and heat transfer (see Figure 1). Heat transfer presents unique challenges for on-body sensors. Choosing appropriate on-body locations and sensor attachments can help address these challenges. Heat transfer can occur from individuals wearing dark clothing or from the wearer’s body heat and perspiration. Hanging a sensor close to the body, without direct contact, is an optimal approach, which is why we wanted to explore earrings as a potential solution. Designing a wearable shield that is small enough to be worn while adequately protecting the sensor from direct sunlight and allowing sufficient airflow was a significant and main challenge for our design. We also investigated various methods for securing the iButton within the shield. The iButton attachment needed to ensure adequate airflow while allowing for easy insertion and removal.

**Figure 2:**
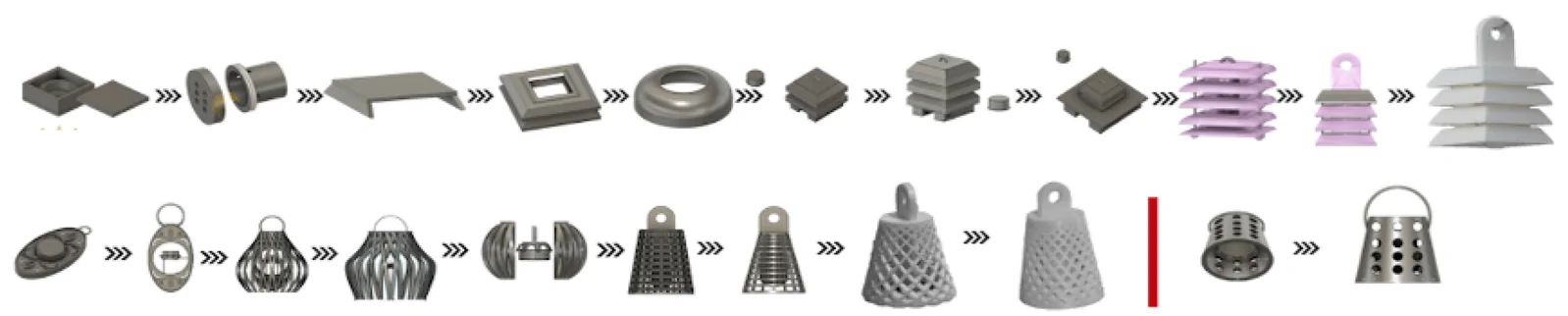
The design process of rapidly prototyping wearable shields.

**Figure 3:**
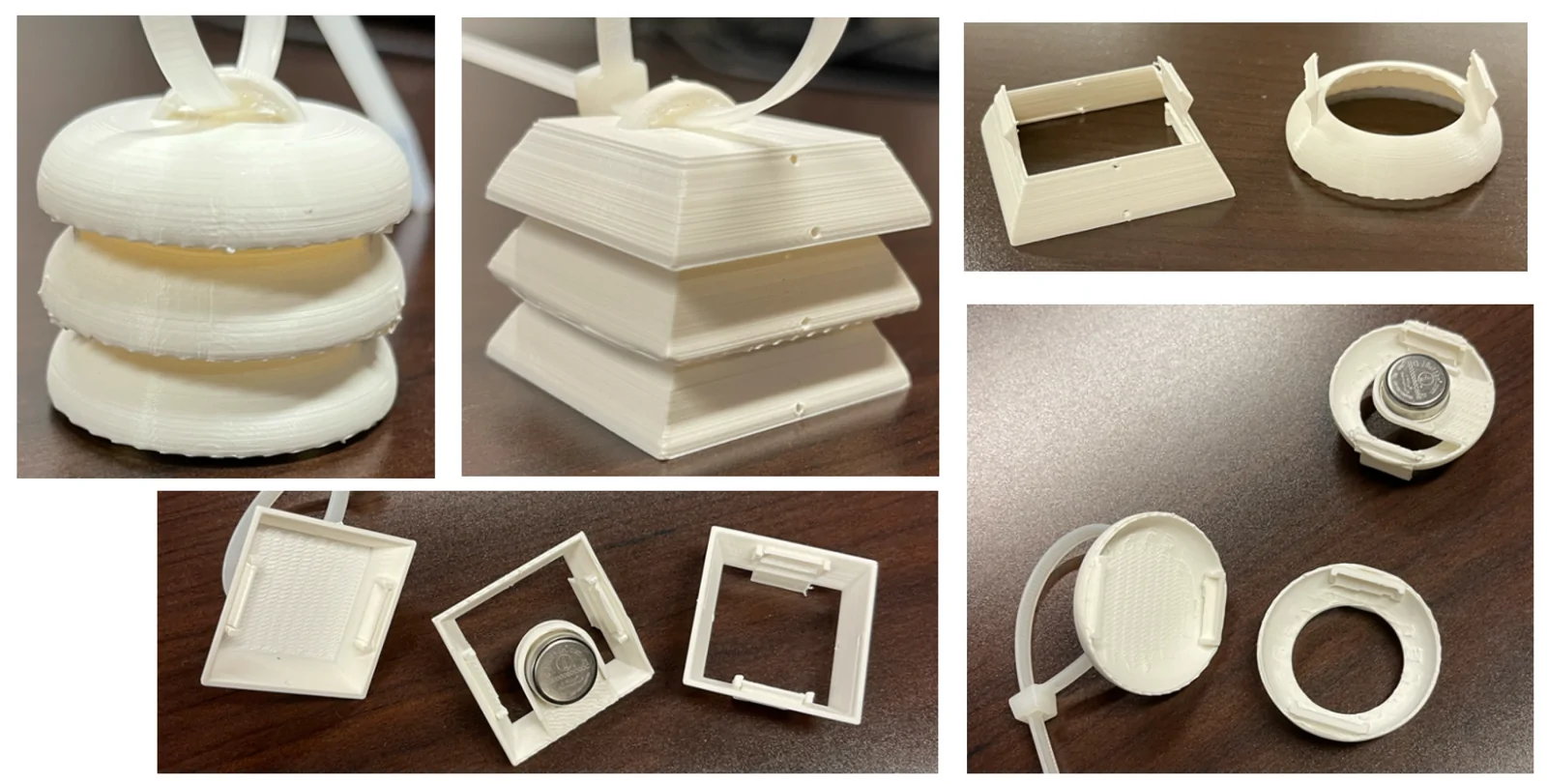
Images of circle and square gill shields assembled and disassembled.

After several prototypes (see Figure 2), we selected two main designs, a gill-plated design and a hoop skirt design. The gill design is based on the design for standard solar radiation shields. The nested layers provide shade and allow for air flow. We developed two gill designs: a circle and square gill shield. The gills were designed to be 3D-printed separately and attached after printing. The middle and bottom gills are interchangeable; therefore, designs can allow for multiple layers to be added and tested. The gills design also allows for the attachment location of the sensor to vary within the gill system. The gill with the sensor attachment can vary and be placed at any level (top, middle, or bottom). We tested our gill designs with the sensor attachment in the middle. Figure 3 shows an assembled round and square wearable gill shield as well as shows their separated components. The gill shields were designed to be worn at the waist and attached to a belt or belt loop.

**Figure 4:**
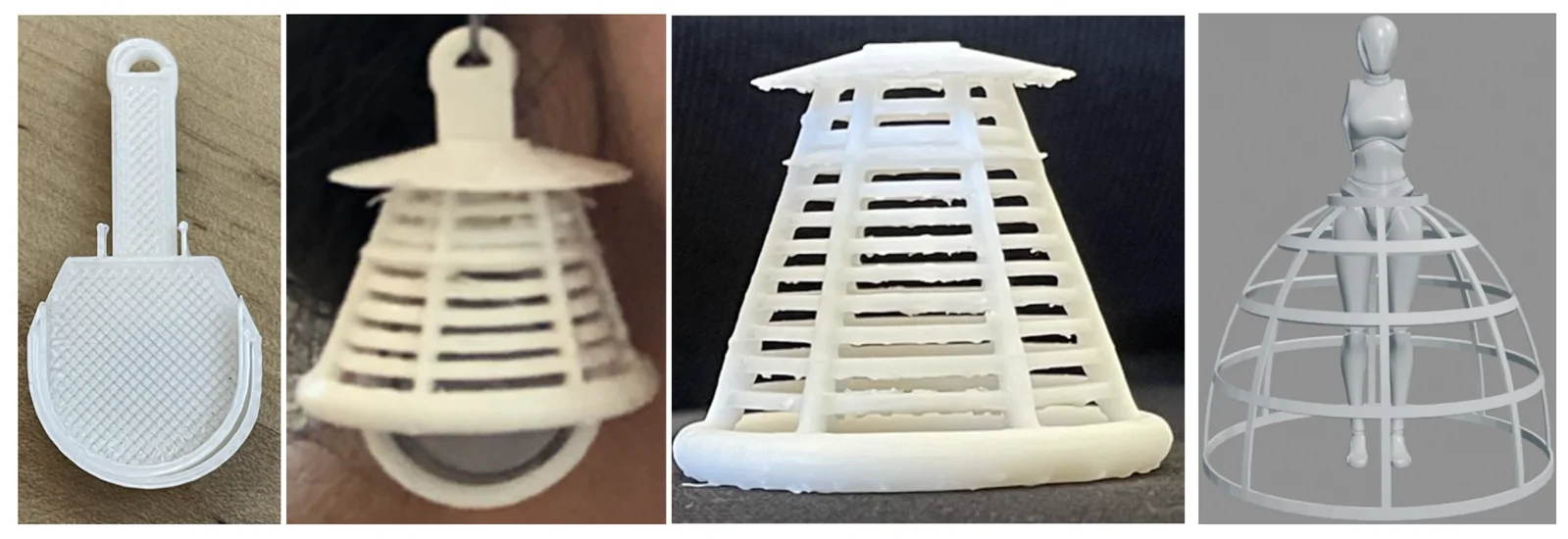
Images of the small and large earring design, the fob for iButton attachment and a picture of the structural design behind a hoop skirt which inspired the design for the earrings.

For earrings, we opted for a different design. The size of the gill-plated shield did not easily scale down to be worn as an earring. Therefore, we explored other design options and selected a design pattern based on the hoop skirt (see Figure 4). This design incorporates vertical structural supports that slant inward with circle horizontal rings attached to the vertical structural support. Decreasing the width of the shield vertically allowed for shields to have a smaller size and weight. For the hoop skirt design, we hang a pendant (fob) in the middle of the hoop skirt which holds the iButton. The pendant is 3D-printed separately from the hoop skirt and is assembled after printing by sliding through a hole at the top of the shield. The pendant can be easily used and exchanged for other designs. In order to explore the size restraints for the earrings, we prototyped two versions: a small earring shield and large earring shield. The large earring has 12 horizontal slats compared to the small earring shield, which has 8 horizontal slats (see Figure 4).

**Figure 5:**
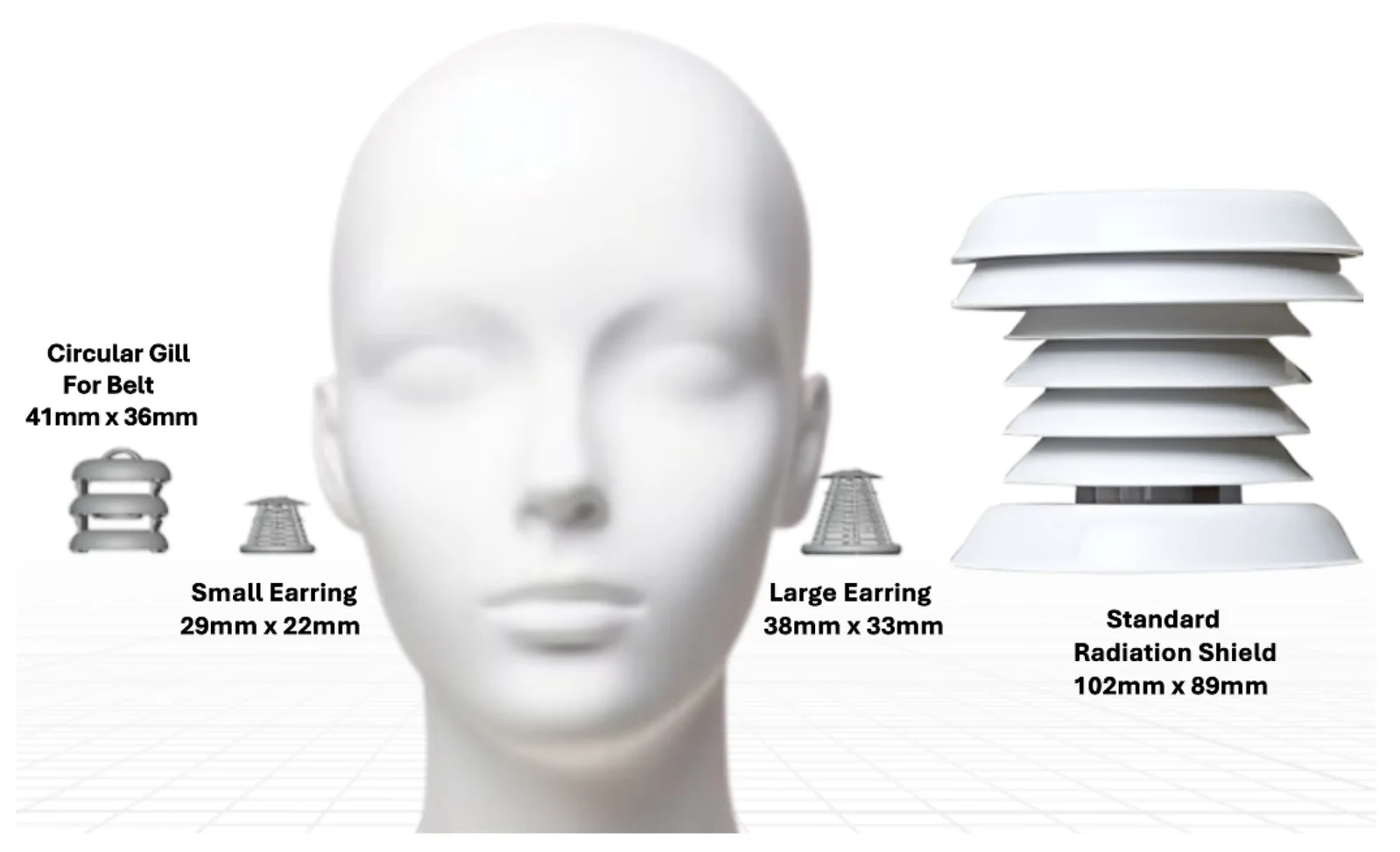
Radiation shield sizes compared with each other along with their dimensions.

Our final design process resulted in four form factors, two for the belt (square and round) and two for the earrings (small and large). Our prototypes for both the belt and earrings had to address the main challenge of scale. Our prototype designs needed to be effective at shielding our sensors from overheating in direct sunlight and simultaneously be small enough in size that they could be worn for everyday use and long-term deployment for different stakeholders. Figure 5 illustrates the size of a typical solar radiation shield (on the right) adjacent to our prototypes to illustrate that standard off-the shelf solar radiation shields are not conducive for everyday wearability.

The wearable solar radiation shields were designed in Fusion 360 and 3D-printed in a makers lab on a university campus. We selected two different materials for our design. We used a filament (Ultimaker Pearl-White PLA) for the gill-plated shields and a resin (Formlabs White Resin v4) for the hoop skirt design. We began printing all prototypes with PLA. To obtain more consistent results with less dedicated time, we switched to stereolithography (SLA) printing with resin for the hoop skirt design. Because the earrings had more intricate designs and elements compared to the belt, the switch to SLA and resin was needed, as SLA printing produces higher-accuracy products. Both materials are white in order to minimize the amount of solar radiation absorbed by the shields when outdoors.

### 2.1. Sensor Details

For our evaluation study, we used two sensor technologies with integrated data-logging features for continuous temperature monitoring. We used an in-situ sensor, MX2302A, (Onset Computer Corporation, Bourne, MA, USA) as a reference point and the iButton sensors as the wearable sensors to be worn with our new solar radiation shields. The MX2302A is a research-grade sensor designed for long-term outdoor deployments, featuring a temperature accuracy of ±0.25 °C and a resolution of 0.02 °C. It is equipped with a standard solar radiation shield that enhances its performance in outdoor environments. In contrast, the DS1923 iButton Hydrochron (Analog Devices, Wilmington, MA, USA) is a compact, coin-sized sensor capable of measuring both temperature and relative humidity with an accuracy of ±0.5 °C and operational modes of 8-bit or 16-bit resolution. The DS1921 iButton Thermochron is similar but only measures temperature and has a slightly lower accuracy of ±1 °C. See Table 1 for technical specifications for each sensor.

Both the MX2302A and iButtons can be programmed to log temperature at user-defined intervals, typically ranging from once per minute to several hours. In this study, all sensors were set to log data every 5 min to ensure consistency with the reference MX2302A sensor. The iButtons contain an integrated temperature sensor, real-time clock, and memory for data storage, allowing them to operate independently. They are programmed using OneWireViewer software prior to deployment. The iButton logging duration is limited to 5 min to conserve battery and memory capacity. With these settings, the sensor can record data for approximately 5.5 days before reaching full capacity.

Data retrieval differs between the two sensors: the MX2302A utilizes HoboConnect, a mobile app for downloading data, while the iButtons’ data are accessed through OneWireViewer. The MX2302A was calibrated by the manufacturer, per their company’s standard practice, before sensor deployment and is reported to have a drift of less than 0.01 °C (0.018 °F) per year. No further calibrations were conducted for the MX2302A as the sensor was deployed one month prior to the evaluation study. For the iButtons, no manual calibration was performed during this study. Instead, software corrections for temperature and humidity were handled automatically by the OneWireViewer software, as specified in the manufacturer’s documentation.

### 2.2. Evaluation Methods

**Figure 6:**
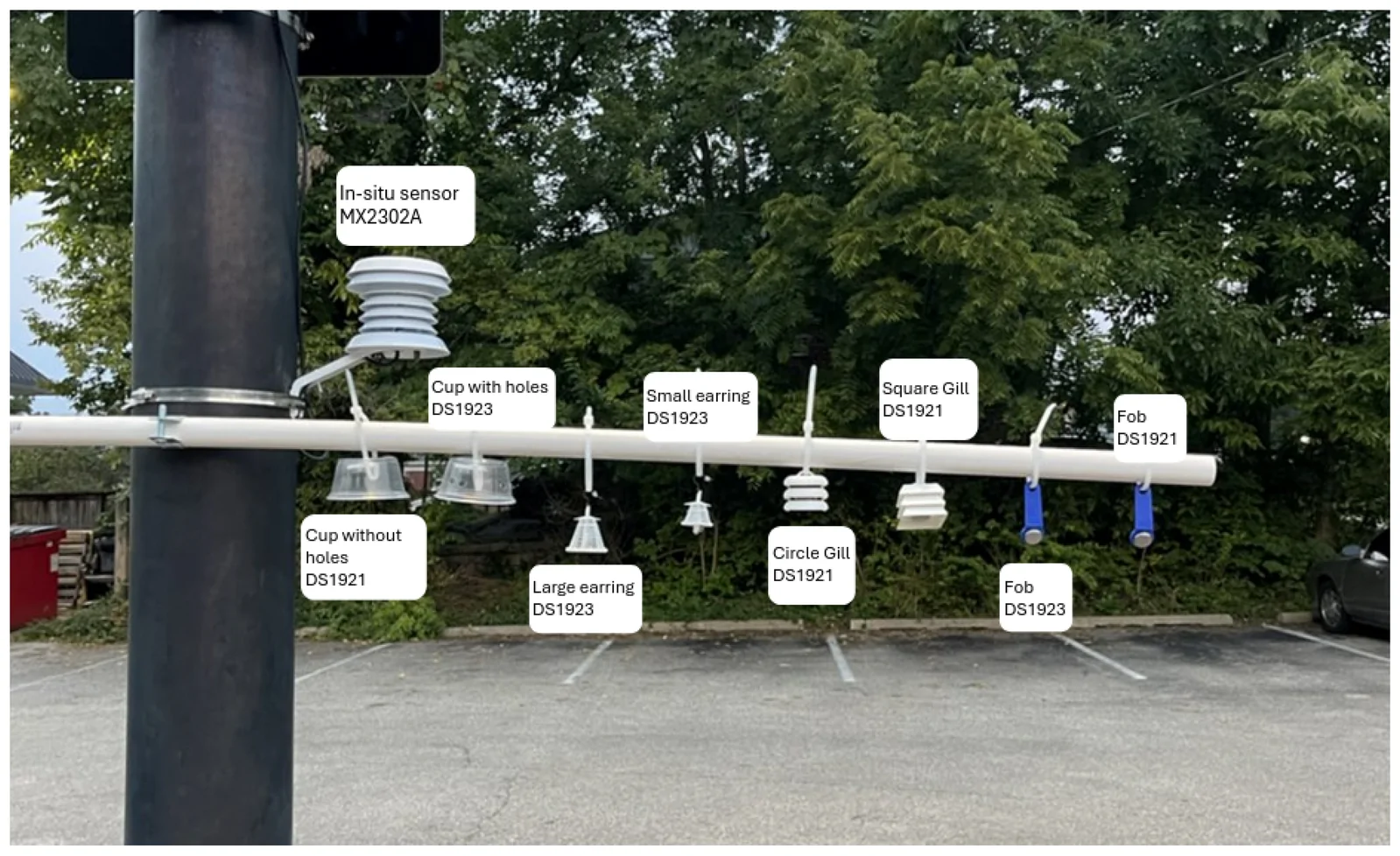
Wearable solar radiation shields deployed in the field next to a research grade MX2302A T/RH sensor. The sensors are described in Table 1.

We next evaluated our designs to investigate whether they reduce temperature bias for the iButton sensors. We deployed the shields housing iButtons in the field by co-locating our wearable solar radiation shields next to a research grade temperature/relative humidity sensor, the MX2302A T/RH sensor. The MX2302A sensor used for our evaluation study is installed in a parking lot and is part of a larger urban sensor network located on the campus of Indiana University (Bloomington, IN, USA). We mounted a white PVC pipe to the same light post holding the MX2302A sensor. From the PVC pipe, we hung four versions of our shield designs using zip ties: circle gill, square gill, small earring, and large earring. We also hung two unshielded iButtons housed in plastic fobs as well as 2 iButtons housed in plastic ketchup cups. One of these cups was perforated with holes. Plastic cups were used in the evaluation, as they are often recommended as a cheap and easy way to shield the iButtons. Even though they have been used in previous studies, ketchup cups tend to overheat iButton sensors instead of reducing their temperature bias [34]. Figure 6 illustrates the evaluation study configuration. The sensors were deployed during the summer on hot days that were ideal for urban heat island conditions (high temperatures, low wind speed, low precipitation and low cloud cover). Deployment occurred from 15 August to 22 August, which was the hottest week of 2023 for Bloomington, IN. Sensors and shields were taken down each day, as the iButtons are not waterproof and not designed for long-term deployments.

The iButtons were manually started using the OneWireViewer software, placed into the radiation shield, set outside each morning with enough time to acclimatize to outdoor conditions, and collected every night to download data. Each iButton was set to record data at the highest resolution available and to start logging data at the nearest five-minute interval, which matched the logging time for the MX2302A sensor.

### 2.3. Bias Temperature Variable

We calculated an error metric to assess the overall bias and accuracy of iButton sensors when housed in our wearable solar radiation shields. To validate the wearable sensors, we calculated difference scores to estimate sensor bias. We calculated the average bias for each wearable shield in relation to the in situ sensor (MX2302A) using the following equation:

$$bias=\frac{1}{n}\sum_{i=1}^{n}\left(s_{i}-o_{i}\right)$$

where *n* is the total number of recorded observations, $s_{i}$ is the recorded temperature for the wearable sensor for observation *i*, and $o_{i}$ is the corresponding in situ sensor observation.

We calculated bias scores for the entire study period (10 a.m.–6 p.m.) as well as during the hottest portion of the day (2 p.m.–6 p.m.). To confirm a statistical difference between the bias values, we ran a one-way ANOVA and employ Tukey’s post hoc pairwise comparisons.

## 3. Results

The performance of different solar radiation shield designs reveal significant variations in temperature bias compared to the reference sensor.

### Temperature Bias Analysis

The bias scores were calculated for each observation across the entire duration of the study period and then averaged for each shield design. Table 2 shows the T-test results conducted between the reference MX2302A sensor and each of the shield designs. All temperature differentials between the reference sensor and the shield designs were statistically significant. Table 2 ranks the performance of the shields based on their mean bias scores, with the circle gill outperforming all other designs with the lowest mean bias value of 1.87 °F. The two earring designs were the next best performing shields. The cup and the fob exhibit a mean bias score that is two times greater than the gill and earring designs. Compared to all other designs, the fob had the highest bias score of 5.66 °F. Results demonstrate that the best performing shield design (circle gill) is able to reduce the heating effect by 3.73 °F compared to the unshielded fobs. Error consistency metrics indicate that earring and gill designs have the lowest bias standard deviation and interquartile ranges (specifically the small earring design), suggesting more consistent performance, while the fob and cup designs show the highest variability. The combination of Cohen’s d and R-squared values presents a broader perspective of the bias: high $R^{2}$ values (circle gill and earrings) suggest that despite the small effect size, the shield’s readings closely follow the reference sensor readings; low $R^{2}$ values (cup and fobs) combined with larger effect size indicate both poor fit and greater deviation from our reference sensor. There is a clear distinction between the high-performing gill and earring designs and the lower-performing cup and fob designs in terms of error spread.

An ANOVA test was performed to assess whether there are statistically significant differences in mean performance across various shield designs. Table 3 presents a selection of these comparisons, highlighting both statistically significant and non-significant results.

A key finding from this analysis is that the shield designs reduce bias scores compared to the cup and fob deployments. The comparisons between shields and cups/fobs consistently show statistically significant differences, whereas the comparisons within the cup and fob categories show no statistically significant differences. The Fob 1921 vs. Cup Holes comparison (F-statistic: 0.0064, *p*-value: 0.9364) and Fob 1921 vs. Cup No Holes (F-statistic: 2.1854, *p*-value: 0.1396) both have *p*-values well above the 0.05 threshold, indicating that these designs perform similarly despite their structural differences. Furthermore, the comparisons within the shield designs themselves (Large Earring vs. Small Earring, Large Earring vs. Circle Gill) show no statistically significant differences (*p*-values > 0.05). This suggests that variations in size among earring designs or the structural differences between earring and gill designs do not lead to significant performance disparities.

#### Temperature Bias Analysis (2 p.m.–6 p.m.)

We also looked at the temperature differences between the shield designs for just the hottest parts of the day (2 p.m.–6 p.m.) (Table 4). The large earring design emerged as the top performer during these high heat hours with a mean bias of just 1.66 °F. This represents an improvement over its overall performance, suggesting it is effective in high-temperature conditions. The circle gill design maintained consistent performance across all times of the day, including the hottest hours with a mean bias of 1.89°F. The difference of 5 °F between the large earring (1.66 °F) and Fob 1923 (6.665 °F) designs shows the difference in their abilities to mitigate the heating effect on temperature sensors.

**Figure 7:**
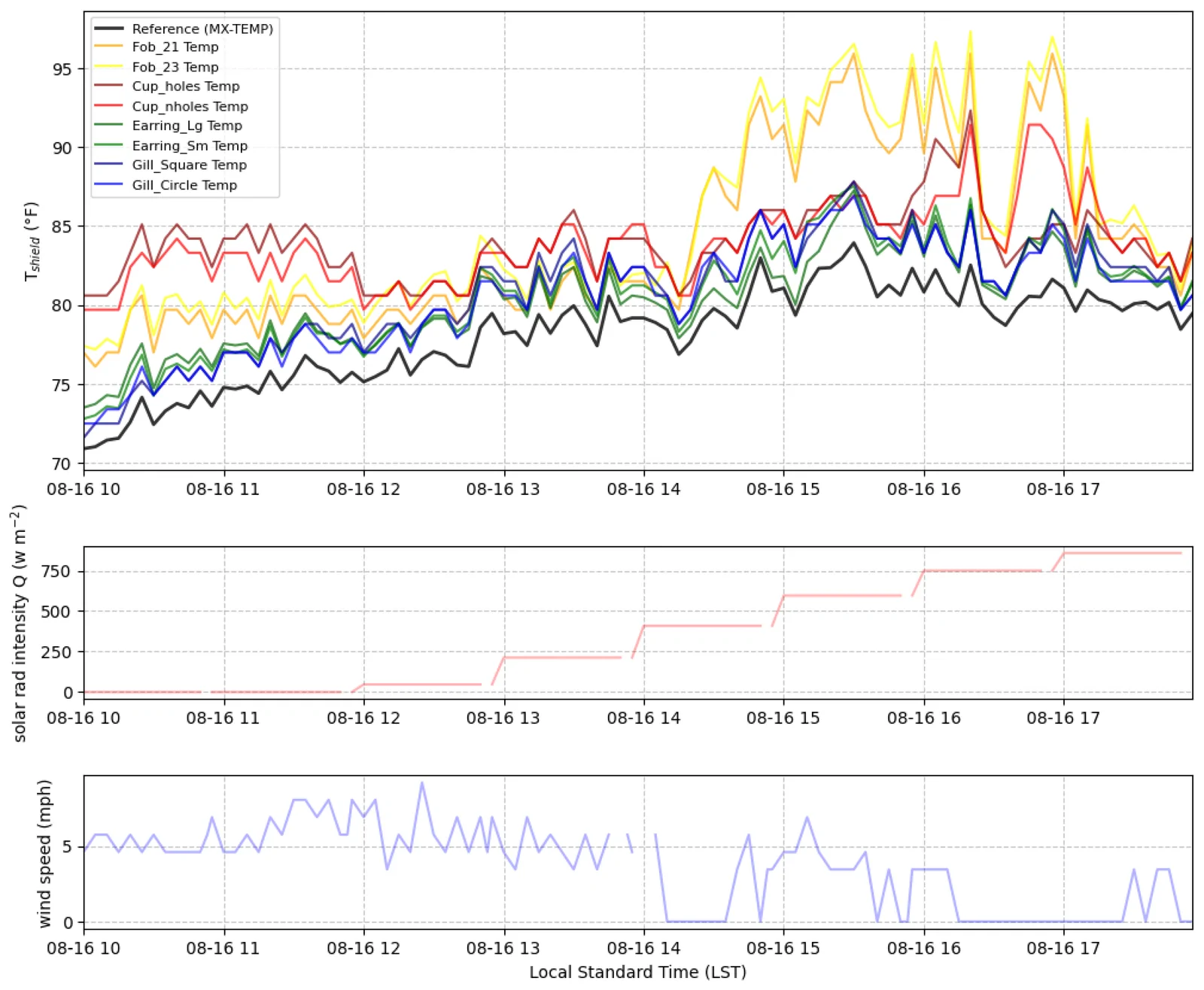
Time series of shielded sensor temperatures, solar radiation intensity, and wind speed throughout the day.

Figure 7 shows the daily performance of each shield design under varying solar radiation and wind conditions. The solar radiation data were obtained from Open-Meteo, which provides high-resolution weather model data [36]. Wind speed was sourced from MesoWest, which aggregates and archives National Weather Service (NWS) data [37]. Both datasets were collected from the Bloomington airport’s weather station (KBMG), which is a standard Automated Surface Observing System (ASOS) NWS station located approximately 5 miles (8 km) from the study site.

In line with the statistical findings, the fob and cup designs display the largest temperature biases with peak temperatures exceeding 105 °F during high solar radiation periods between 2 p.m. and 5 p.m. At these times, solar radiation intensity rose sharply to peak levels, while wind speed dropped to near zero. This combination led to pronounced temperature spikes in the fob and cup designs, diverging significantly from the reference sensor. In contrast, the gill and earring shields maintained stable temperatures, closely matching the reference MX sensor despite the high solar radiation and lack of wind. This stability suggests that these designs offer superior protection from direct sunlight even during intense radiation.

## 4. Discussion

All four of our wearable solar radiation shields significantly reduced the bias scores for the iButtons by approximately 3 °F on average, thereby reducing more than 50% of the original bias score from the iButtons. This result illustrates that our wearable shield designs can increase the performance for the most popular wearable ambient temperature sensor currently used in urban heat research studies. All four shield designs performed relatively similarly in their reduction of bias scores. They exhibited a 0.3 °F difference across the four shield designs, but none of these difference scores were statistically significant. The circle gill shield performed the best with a 1.87 °F bias score as compared to the unshielded iButton 1923 with a 5.66 °F bias score. The circle gill shield is the closest approximation to a standard solar radiation shield, and this may be a reason for its optimal performance. Not only do we see statistically significant bias reduction, but we also see that when iButtons are housed within our wearable shields, they have less variance in their difference scores. Our wearable shield designs are helping to keep iButton temperatures relatively stable when compared to the MX2302A sensor. A previous study by Habeeb et al. [18] showed that when iButtons were worn unshielded in direct sunlight, their bias scores would spike dramatically due to overheating from being in the sun. Our shield designs are keeping iButton temperature readings consistent and limiting the overheating of these sensors. The low variance scores and consistent readings may allow for adjustment scores to be applied to the iButtons when worn in a wearable shield. More investigation is needed to see if there is a pattern in the variance of the data, such as if higher variances occur during certain periods of the day (morning vs. afternoon) or during specific climatic conditions (hot, clear skies, low wind). This could allow for a better tailoring of adjustment scores. Our field deployment evaluation is the first step toward illustrating the potential for solar radiation shields to be designed at a wearable scale and them to be integrated as accessory for wearable sensors.

### 4.1. Challenges

We encountered several challenges when designing and prototyping our solar radiation shields. Our first challenge was to design a system that could be comfortably worn while at the same time protect sensors from overheating. We decreased the shield sizes so they could be easily attached to a belt, worn as earrings, and house the iButton sensor within the shield. The sensor attachment designs took several iterations. The iButton sensors needed to be easy to remove from the shield, as remote data access is not possible with the iButton. We designed the sensor attachment to maximize air circulation around the sensor while simultaneously limiting the amount of material in direct contact to reduce the possibility of heat transfer. The modular design of the gill shield provided for flexibility of configuration and also aided with sensor removal, but it was challenging to develop gills and tab attachments that did not break during assembly and disassembly due to the brittleness of the material. The hoop skirt design for the earrings was challenging, as we had to balance printing optimization with the design criteria. For example, a lot of post-processing was needed for the earrings, since they are designed as a whole system with air slots.

There is a need for advancement in technology for wearable sensors for environmental monitoring, especially for temperature. Although the iButton is the most utilized wearable temperature sensor, it has a lot of drawbacks. These drawbacks include difficulty with data access, data storage limitations, needing specialized software and hardware for data downloads and accessing sensor settings. Real-time data access is not possible with the iButton; therefore, the ability to design applications delivering real-time heat exposure data to stakeholders is not feasible with the iButton.

We could not easily explore other design augmentations such as integrating the ability to aspirate the shields. There are two types of solar radiation shields: passive ventilation shields which rely on their physical design to allow air flow, and active ventilation shields which rely on fans to enhance air circulation. Small wearable solar radiation shields may benefit from active ventilation designs but require advanced engineering that is not yet widely available. For example, there needs to be a fan with a small footprint and power supply to limit overall weight while offsetting negative effects from waste heat.

### 4.2. Next Steps

A wearable evaluation study similar to Habeeb et al. [18] is needed to investigate how these wearable solar radiation shields perform when worn on-body in the built environment. Further research is needed to explore how individual differences such as age, gender, race, hair color and even perspiration from activities may impact wearable shield performance. Clothing and hairstyles may also impact performance. For example, wearing black pants or shorts may impact the gill shields when worn on the waist. Someone with long dark hair wearing their hair down, as compared to up in a pony tail, may also impact earring performance. If sensors are worn in both ears, then there is a strong likelihood that sensors will have different readings depending on which sensor is facing the sun. An additional evaluation study will help tease out these issues. Other on-body locations could be explored such as integrating sensors into hats or helmets (such as when they are needed for occupational reasons). Locating sensors above the head and detached from the body has the possibility of being a reliable on-body location by limiting heat transfer from the body. Integrating sensors into everyday accessories, especially those used during high heat conditions, can facilitate the adoption and use of the technology.

**Figure 8:**
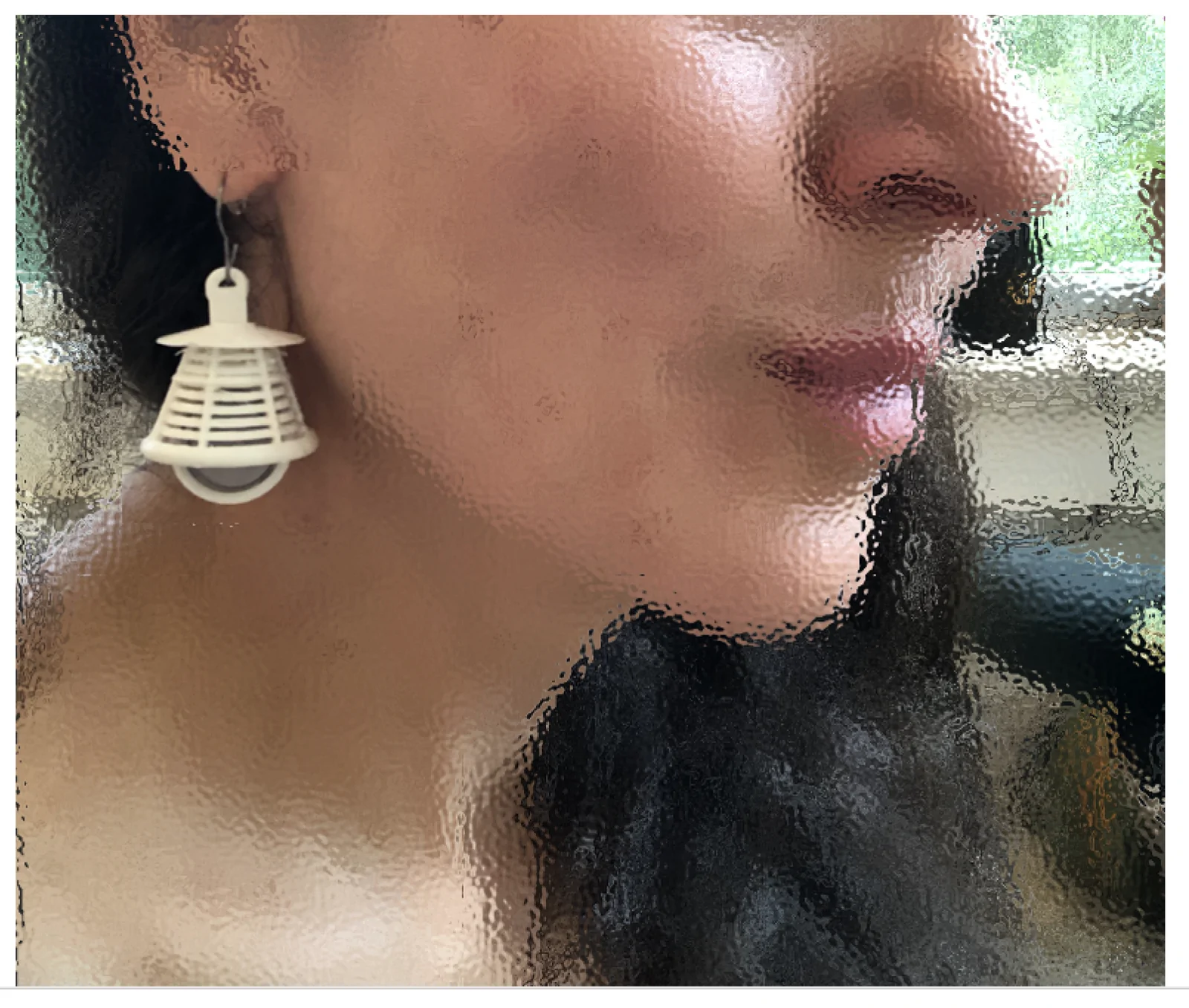
Image of the earring shield being worn. A foray into fashion culture with wearable sensors.

Integrating wearable sensors into fashionable accessories, such as jewelry, presents an innovative opportunity to enhance user adoption and enable continuous environmental monitoring. Wearable technology has already evolved into the fashion industry [38]. Exploring how to evolve wearable environmental sensors to actively participate in the culture of fashion is a great opportunity both to advance the design of sensors and to integrate them into everyday lives instead of being relegated solely for research studies. Jewelry is a prime and rapidly evolving design space for emerging trends for wearable technology [39,40,41,42]. Earrings, as a design style for our radiation shields, were not only selected due to their potential as an optimal on-body location but also to explore the potential of a design artifact linking jewelry, fashion, and technology (see Figure 8).

Evolving fashion trends may include summer jewelry such as smart earrings for extreme heat detection and may include other form factors such as charms for bracelets and belts. Extreme heat is often overlooked as a serious threat to personal health, which is why it is often called a *silent killer*. Integrating sensing tools for heat detection with jewelry and fashion could be an effective way to promote heat awareness and support positive and life-protecting behavior change.

## 5. Conclusions

Current wearable temperature sensors have been shown to overheat when worn in direct sunlight and in areas with a high percentage of impervious surfaces, such as parking lots and big box developments. In this study, we designed and prototyped wearable 3D-printed solar radiation shields in order to improve the accuracy of wearable temperature sensors. Our wearable solar radiation shields are designed specifically for the iButton due to its popularity in the research community and due to its small size. Our wearable shields are designed for the belt and to be worn as earrings. This is the first study to prototype a wearable solar radiation shield and the first study to use the earring as an on-body location for capturing ambient temperatures in urban environments. We conducted an initial validation of our designs by deploying them in the built environment. The evaluation showed that our shields on average reduced bias scores by approximately 3 °F, representing a 50% reduction in unshielded iButton bias scores. Our study demonstrates the potential for solar radiation shields to be designed at a wearable scale and integrated as accessories for wearable temperature sensors. Our shields can help to improve the accuracy of data collection for person-based environmental sensing in urban settings when measuring extreme heat.

## Figures and Tables

**Table 1:** Technical specifications of sensors used in this study.

Sensor Model–Vendor	Parameters Monitored	Range	Accuracy	Resolution	Drift	Response Time
MX2302A Onset	Temperature	−40 to 70 °C (−40 to 158 °F)	±0.25 °C from −40 to 0 °C (±0.45 °F from −40 to 32 °F)	0.02 °C (0.036 °F)	<0.01 °C (0.018 °F) per year	~3 min
	Relative Humidity	0–100% RH, −40 to 70 °C (−40 to 158 °F)	±2.5% from 10% to 90%	0.01%	<1% per year	~15 s
DS1923 iButton Hygrochron- Analog Devices	Temperature	−20 to 85 °C (−4 to 185 °F)	±0.5 °C from −40 to 0 °C (± from −40 to 32 °F)	8-bit mode: 0.5 °C; 16-bit mode 0.0625 °C (0.036 °F)	130 s	N/A
	Relative Humidity	0–100% RH	±0.5%RH	8-bit mode: 0.64%RH; 12-bit mode: 0.04%RH	30 s	<1% per year
DS1921G-F5 iButton Thermochron-Analog Devices	Temperature	−40 to 85 °C (−40 to 185 °F)	±1 °C from −30 to 70 °C (± from −22 to 158 °F)	0.5 °C	130 s	NA

**Table 2:** Performance metrics of temperature bias for various shields relative to the refernce sensor (MX2302A).

Shield	Mean Bias (°F)	Std Dev (°F)	IQR * (°F)	RMSE * (°F)	$R^{2}$ *	t-Stat	*p*-Value	Cohen’s d
Circle Gill	1.8721	0.8105	1.1575	2.0397	0.9281	−55.7225	0	0.2404
Large Earring	1.9557	0.8625	1.2658	2.1371	0.9211	−54.7043	0	0.2533
Small Earring	2.0344	0.7374	0.9315	2.1637	0.9191	−66.5575	0	0.2621
Square Gill	2.1752	1.0458	1.6075	2.4131	0.8994	−50.1799	0	0.2781
Cup with no holes	4.5273	2.8225	3.9250	5.3337	0.5086	−38.6960	0	0.5632
Fob 1921	4.7747	2.8874	3.8750	5.5786	0.4624	−39.8930	0	0.5716
Cup with holes	4.7870	2.3766	3.1150	5.3436	0.5067	−48.5929	0	0.6005
Fob 1923	5.6630	3.0131	3.8988	6.4135	0.2894	−45.3412	0	0.6757

* IQR = interquartile range, RMSE = root mean square error, $R^{2}$ = coefficient of determination.

**Table 3:** Selected ANOVA results.

Comparison	F-Statistic	*p*-Value (*p* < 0.05)
Fob 1921 vs. Cup Holes	0.0064	0.9364
Fob 1921 vs. Cup No Holes	2.1854	0.1396
Large Earring vs. Small Earring	2.8023	0.0944
Cup Holes vs. Cup No Holes	2.8852	0.0897
Large Earring vs. Circle Gill	2.9045	0.0886
Fob 1921 vs. Small Earring	492.0842	0
Fob 1923 vs. Small Earring	796.3439	0
Fob 1921 vs. Circle Gill	545.1739	0
Fob 1923 vs. Circle Gill	859.0954	0
Cup No Holes vs. Small Earring	424.9752	0
Cup Holes vs. Circle Gill	784.3122	0
Cup Holes vs. Small Earring	712.1606	0
Cup No Holes vs. Circle Gill	475.8060	0

**Table 4:** Performance metrics of temperature bias for various shields relative to the refernce sensor (MX2302A) between 2 p.m. and 6 p.m.

Shield	Mean Bias (°F)	Std Dev (°F)	IQR * (°F)	RMSE * (°F)	$R^{2}$ *	t-Stat	*p*-Value	Cohen’s d
Large Earring	1.6675	0.8376	1.0885	1.8653	0.9370	−34.1362	0	0.2186
Circle Gill	1.8996	0.9131	1.5125	2.1070	0.9196	−35.6704	0	0.2488
Small Earring	2.0182	0.8729	1.1877	2.1983	0.9125	−39.6430	0	0.2647
Square Gill	2.1904	1.0659	1.6800	2.4352	0.8926	−35.2369	0	0.2855
Cup with No Holes	3.0322	1.6363	1.6375	3.4442	0.7852	−31.7751	0	0.3893
Cup with Holes	3.8067	1.8293	1.9075	4.2221	0.6771	−35.6820	0	0.4816
Fob 1921	5.7537	3.4811	6.1025	6.7217	0.181703	−28.3399	0	0.6868
Fob 1923	6.6682	3.6654	6.5655	7.6062	−0.047811	−31.1932	0	0.7929

* IQR = interquartile range, RMSE = root mean square error, $R^{2}$ = coefficient of determination.

## Data Availability

The original contributions presented in this study are included in the article. Further inquiries can be directed to the corresponding author.

## Abbreviations

The following abbreviations are used in this manuscript:

FDM	Fused Deposition Modeling
PLA	Polylactic acid
PVC	Polyvinyl chloride
SLA	Stereolithography
T/RH	Temperature/Relative Humidity
